# Sexual dimorphism in PAR_2_-dependent regulation of primitive colonic cells

**DOI:** 10.1186/s13293-019-0262-6

**Published:** 2019-09-06

**Authors:** Julie Noguerol, Pierre-Jean Roustan, Mikael N’Taye, Léo Delcombel, Corinne Rolland, Laura Guiraud, David Sagnat, Anissa Edir, Chrystelle Bonnart, Alexandre Denadai-Souza, Céline Deraison, Nathalie Vergnolle, Claire Racaud-Sultan

**Affiliations:** IRSD, Université de Toulouse, INSERM, INRA, ENVT, UPS, CHU Purpan, place du Dr Baylac, 31024 Toulouse Cedex 3, Toulouse, France

**Keywords:** Colon primitive cells, Sexual dimorphism, Protease-activated receptor

## Abstract

**Background:**

Sexual dimorphism in biological responses is a critical knowledge for therapeutic proposals. However, gender differences in intestinal stem cell physiology have been poorly studied. Given the important role of the protease-activated receptor PAR_2_ in the control of colon epithelial primitive cells and cell cycle genes, we have performed a sex-based comparison of its expression and of the effects of PAR_2_ activation or knockout on cell proliferation and survival functions.

**Methods:**

Epithelial primitive cells isolated from colons from male and female mice were cultured as colonoids, and their number and size were measured. PAR_2_ activation was triggered by the addition of SLIGRL agonist peptide in the culture medium. PAR_2_-deficient mice were used to study the impact of PAR_2_ expression on colon epithelial cell culture and gene expression.

**Results:**

Colonoids from female mice were more abundant and larger compared to males, and these differences were further increased after PAR_2_ activation by specific PAR_2_ agonist peptide. The proliferation of male epithelial cells was lower compared to females but was specifically increased in PAR_2_ knockout male cells. PAR_2_ expression was higher in male colon cells compared to females and controlled the gene expression and activation of key negative signals of the primitive cell proliferation. This PAR_2_-dependent brake on the proliferation of male colon primitive cells was correlated with stress resistance.

**Conclusions:**

Altogether, these data demonstrate that there is a sexual dimorphism in the PAR_2_-dependent regulation of primitive cells of the colon crypt.

**Electronic supplementary material:**

The online version of this article (10.1186/s13293-019-0262-6) contains supplementary material, which is available to authorized users.

## Background

Cells in the different adult organs have a sexual identity influencing their behavior in physiology and pathophysiology [1]. For instance, neuronal survival is differently regulated between male and female developing brain in a hormone-independent way [2]. Furthermore, adult stem cells also display sexual differences in the responses to growth factors and cytokines [3]. Despite the implication of energetic and proliferative pathways, the mechanisms supporting this sexual dimorphism remain to be better understood.

We previously demonstrated that cell adhesion is implicated in the sexually dimorphic survival of leukemic stem cells [4]. Downstream of integrin engagement, Akt-dependent pathways were shown to control the survival of male leukemic stem cells, whereas opposite GSK3β-dependent pathways were required for females. This sexual dimorphism also influenced stem cell clonogenicity capacities and resistance to chemotherapy [4]. Importantly, the dependency on either GSK3 or Akt pathways was switched in normal adherent male and female hematopoietic stem cells, respectively. This indicates the occurrence of plasticity in these sex-related signaling pathways [4].

Recently, Hudry and co-workers have shown that intestinal stem cells (ISC) in adult drosophila display a sexual dimorphism [5]. A master gene in sexual development and dosage compensation, *Sxl*, was found to control higher proliferative capacities of female ISC in gut homeostasis and regeneration. A large genetic investigation indicated that cell-intrinsic mechanisms such as carbohydrate metabolism and oxidation-reduction processes in males and cell cycle process in females were implicated in those sex differences. Interestingly, two genes were specifically found as positive regulators of the proliferation in female intestinal progenitors: a growth factor (*imaginal disc growth factor 1*) and an anti-protease (*serpin 88Eb*).

The dialogue between stem cells and their microenvironment is crucial for intestinal crypt homeostasis. Growth factors and proteases are key regulators of pro-liferation and differentiation of primitive cells, including stem cells and progenitors [6]. Indeed, we found expression of protease-activated receptors (PAR) PAR_1_ and PAR_2_ along the colon crypt and demonstrated that PAR_2_ plays a critical role in the survival of primitive cells cultured in 3D as colonoids [7]. Interestingly, the pro-survival role of PAR_2_ was dependent on the activation of GSK3β in a β-arrestin 2 complex and was associated with inhibition of cell proliferation. On the other hand, PAR_1_ activation triggered Akt activation and colonoid growth [7]. Furthermore, we have shown that PAR_1_ is implicated in the maturation and apoptotic behavior of primary colonoids treated by thrombin [8].

PAR expression is upregulated in digestive pathologies such as inflammation and cancer [9]. Moreover, a sexual dimorphism has been described in human digestive pathologies, both in incidence and localization, indicating a poorer outcome for male patients developing inflammatory bowel diseases and colorectal cancer [10, 11]. Interestingly, sexually dimorphic genes in prepubescent mouse intestine and colon are mostly linked to inflammation and cancer [12]. If this sexual dimorphism is related to PARs is unknown, and thus, it is crucial to get a better knowledge on the role of PARs in crypt homeostasis and their sex-dependent regulation.

Here, we investigated a potential sexual dimorphism in PAR_2_-dependent regulation of ISC. Indeed, the PAR_2_-GSK3β pathway controlling ISC survival may pave the way to inflammation and cancer where GSK3β is overactivated [13]. Further, PAR_2_ activity is known to control the expression of cell cycle genes [14] and has been shown to display sexual dimorphism in vasodilatation and pruritus [15, 16]. The primary organoid model was chosen to investigate a potential sexual dimorphism in PAR_2_-dependent regulation of ISC. Indeed, in that culture conditions, isolated stem cells need to cope with stress and specific sex-related mechanisms can be highlighted [17]. Importantly, the stress-induced mechanisms control further lineage fidelity in tissue repair [18]. We first evaluated the survival and proliferative capacities of stem cells and progenitors from murine male and female colons in the primary organoid model. Secondly, we measured the impact of PAR_2_ activation or knockout and related molecular pathways on those colonoids.

## Methods

### Antibodies and pharmacological inhibitors

Monoclonal antibodies: CD44 clone IM7 (Biolegend, Ozyme, Saint Quentin Yvelines, France; used at 1/200); Ki67 clone SP6 (Abcam, Paris, France; used at 1/500); GSK3β clone 7 (BD Transduction Laboratories; used at 1/2000). Polyclonal antibodies: PAR_2_ antibody was from Santa Cruz Biotechnology (Dallas, TX, USA; used at 1/100); P(Ser21/9)GSK3 (Cell Signaling Technology, Ozyme, Saint Quentin Yvelines, France; used at 1/50 for immunofluorescence and 1/1000 for Western blot); Alexa Fluor 488- and Alexa Fluor 555-conjugated secondary antibodies (Invitrogen Molecular Probes, Thermo Fisher Scientific, Illkirch, France; used at 1/1000). Pharmacological inhibitors: GSK3 inhibitor SB-216763 was from Tocris Bioscience (RD Systems, Lille, France); Rho kinase inhibitor Y-27632 was from Sigma (Saint-Quentin Fallavier, France).

### Animals

C57BL/6 mice deficient for PAR_2_ [19] and the WT littermate C57BL/6 mice were maintained in the animal facilities (Anexplo platform, UMS US006/INSERM, Toulouse, France) under SPF conditions. Animals were maintained in ventilated cages (five mice per cage) in a specific pathogen-free room at 20–24 °C and relative humidity (40–70%) with a 12-h light/dark cycle and given free access to food and water. All animal experiments were conducted in accordance with the Guide for the Care and Use of Laboratory Animals of the European council and were reported in accordance with the ARRIVE guidelines.

PAR_2_^+/−^ mice were inter-crossed to obtain littermates of WT and KO genotypes. Six to ten weeks male and female mice were used in the experiments, and animals from both sexes with the same age were used simultaneously. Animals were euthanized for abdominal laparotomy and colon sampling by a lethal overdose of pentobarbital i.p. followed by cervical dislocation.

### Colonoids and PAR_2_ stimulation

Colon crypts were isolated from the 2/3 ends of descendant colon of C57BL/6 male or female mice, WT, or PAR_2_ KO (*n* = 13 experiments, each including the 4 genotypes, 2–3 mice pooled/phenotype). The colons were opened longitudinally, washed in phosphate-buffered saline (PBS), and incubated in PBS with EDTA (3 mM) and Y-27632 (10 μM) at 4 °C for 10 min, under orbital shaking. Then, colons were gently shaken manually for 2 min at room temperature before incubation in 1 ml DMEM F12 (Gibco, Thermo Fisher Scientific) with collagenase (C6885 Sigma, 5 mg/ml) for 5 min at 37 °C with periodic gentle shaking. Colons were then washed in cold PBS and transferred in PBS with EDTA 10 mM at 4 °C for 10 min, under orbital shaking. After transfer in cold PBS, colons were shaken vigorously for 2 min to isolate crypt fragments. Note that in some experiments (such as for cell sorting, see the paragraph below), male or female crypts have been also isolated by 75 min orbital shaking of colons at room temperature in PBS with 9 mM EDTA plus 3 mM dithiothreitol and 10 μM Y-27632, followed by manual shaking for 2 min in PBS with 10 μM Y-27632. Crypts were pelleted (43 g, 5 min), processed for transcriptome analysis, or resuspended in Matrigel for organoid culture.

One thousand bottom crypts were embedded in 25 μl Matrigel (EHS sarcoma tumor matrix, growth factor reduced, phenol red free, BD Biosciences) and seeded in 48-well plates or 8-well Lab-Tek (Thermo Fisher Scientific). Ten minutes after initiation of Matrigel polymerization at 37 °C, 250 μl DMEM F12 supplemented with 100 U/ml penicillin/streptomycin, 10 mM Hepes, 2 mM Glutamax, N2 (1/100), B27 (1/50) (all from Thermo Fisher Scientific), 100 ng/ml Wnt3a (RD Systems), 50 ng/ml EGF (Gibco, Thermo Fisher Scientific), 100 ng/ml noggin (Peprotech, Neuilly sur Seine, France), and 1 μg/ml R-spondin 1 (RD Systems) was added. It should be noted that N2 and B27 additives contain progesterone and corticosterone and that DMEM F12 was used with phenol red since preliminary experiments have shown no difference in colonoid growth with or without this pH indicator (Additional file 1).

Obtained colonoids in two wells by experimental condition were observed daily using an Apotome microscope (Zeiss Axio-observer, HXP120) to follow their growth. Forty-eight hours after seeding, 3D cultures showed round shape structures whose size increased until the seventh day, when cultures were stopped. Medium was changed every 2 days. In some assays, colonoids were passaged at day 7 of culture through re-embedding in fresh Matrigel. For passage, colonoids were incubated for 30 min with Cell Recovery Solution (BD Transduction Laboratories, BD Biosciences, Le Pont de Claix, France) on ice. This step allows the dissociation of colonoids from Matrigel. Then, the entire colonoids from duplicate wells were pooled and gently re-suspended in ice-cold bovine serum albumin (BSA)-coated tubes containing DMEM F12 supplemented with Hepes, Glutamax, and penicillin/streptomycin as described above. After centrifugation (43 g, 10 min), colonoids were re-embedded in Matrigel and cultured in duplicate wells as described above.

PAR_2_ activation was triggered by a specific agonist peptide SLIGRL from GenScript. One hundred micromolar agonist peptide or its reversed sequence used as control (GenScript or Ezbiolab Inc., Carmel, IN, USA), both dissolved in HBSS, were added to the colonoids every day from 48 h of seeding. At day 6, colonoids were counted at the microscope. Spheroid counting was conducted through bright field microscopy, and for each well of culture, four quadrants were analyzed along the entire depth of the Matrigel layer. The size of colonoids was evaluated after importation of apotome images into the Image J software.

### Reverse transcriptase-polymerase chain reaction (RT-PCR)

Isolated crypts were conserved at − 80 °C in RP1 buffer (Macherey Nagel) until RNA extraction. Total RNAs from 1 × 10^5^ crypts were extracted using the NucleoSpin® RNA/Protein kit (Macherey Nagel) according to the manufacturer’s instructions, including a DNAse (RNAse free) treatment 15 min at room temperature on column. Nucleic acid quantification and purity were assessed by the absorbance A_260_ and the ratio A_260_/A_280_, respectively (Nanodrop 2000, Thermo Fisher Scientific). One microgram of RNA was reverse-transcribed in 20 μl reaction volume using the Maxima first strand kit and following the manufacturer’s instructions (Fermentas, Thermo Fisher Scientific). Quantitative PCR was prepared with LightCycler 480 DNA SYBR Green I Master reaction mix (Roche, Mannheim, Germany), and 15 ng cDNA was used as template for amplification (40 cycles, 60 °C) using 0.6 μM specific primers (Table 1). The run was performed in two technical replicates on a LightCycler 480 Instrument (Roche). All primers used have PCR efficiency > 90%. *Hprt* and *Gapdh* were used as reference genes since these genes have already been used in experiments where PAR_2_ or GSK3 expression/activity varied [15, 20–22]. The delta Ct was calculated (Microsoft Excel software) from the means of reference gene and target gene duplicates. DdCt was used to perform comparisons between male and female or between PAR_2_ WT and PAR_2_ KO tissues. Comparative data shown were calculated with *Hprt* as reference gene, and similar data were obtained with *Gapdh* as reference gene.

**Table 1:** Oligonucleotides used for quantitative RT-PCR. Official gene symbols, NCBI accession number of targeted transcripts, and forward and reverse oligonucleotide sequences are depicted

Genes	NCBI accession number	Forward Reverse
*F2rl1* (PAR_2_)	NM_007974.4	GGACCGAGAACCTTGCAC GAACCCCTTTCCCAGTGATT
*F2r* (PAR_1_)	NM_010169.4	CAGCCAGAATCAGAGAGGACAGA TGTATTTTCACTGGGATTCCTTAGAA
*Ctnnb1*	NM_007614.3 NM_001165902.1	GCTATTCCACGACTAGTTCAG GGAATGGTATTGAGTCCTCG
*Adam10*	NM_007399.4	GAAGATGGTGTTGCCGACAG TTTCCATACTGACCTCCCAGC
*Cd44*	NM_009851.2 NM_001039150.1 NM_001177785.1 NM_001177786.1 NM_001177787.1	TCTGCCATCTAGCACTAAGAGC GTCTGGGTATTGAAAGGTGTA
*Cd24a*	NM_009846.2	GGCACTGCTCCTACCCACGC CACCCCCTCTGGTGGTAGCG
*Dclk1*	NM_019978	CTGCAGCAGGAGTTTCTGTA CCGAGTTCAATTCCGGTGGA
*Myc*	NM_010849.4 NM_001177353.1	AGTGCTGCATGAGGAGACAC GCCTCTTCTCCACAGACACC
*Ccnd1*	NM_007631.2	TGCGTGAGAAGGAGATTGT CTTCGCACTTCTGCTCCTCA
*Rhob*	NM_007483	TGACTTGGGGTCGAGAGGAA AAAAGTGACCCCACTGCACA
*Mapk3*	NM_011952.2	CACTGGCTTTCTGACGGAGT GGATTTGGTGTAGCCCTTGGA
*Sox9*	NM_011448	GAGCCGGATCTGAAGAGGGA GCTTGACGTGTGGCTTGTTC
*Timp1*	NM_001044384.1 NM_011593.2 NM_001294280.2	GAGCCCTGCTCAGCAAAGAG GGACCTGATCCGTCCACAAAC
*Itga3*	NM_013565.3 NM_001306162.1	GATTCCTGGTGGTGAAGGAGG GGGACACAGGTACACAGCAC
*Dusp6*	NM_026268.3	CAAGCAAATTCCTATCTCGG GTCGTAAGCATCGTTCATG
*Itga6*	NM_008397.4 NM_001277970.1	CTCCCTCTCAGACTCGGTCA CTGGCGGAGGTCAATTCTGT
*Timp2*	NM_011594.3	CAACAGGCGTTTTGCAATGC ATCCTCTTGATGGGGTTGCC
*Gsk3b*	NM_019827.7 NM_001347232.1	TGGTGCTGGACTATGTTC GTTCTGTGGTTTAATGTCTCG
*Hprt1*	NM_013556	TCAGTCAACGGGGGACATAAA GGGGCTGTACTGCTTAACCAG
*Gapdh*	NM_001289726	AGGTCGGTGTGAACGGATTTG TGTAGACCATGTAGTTGAGGTCA

### Immunostaining

Histological sections from frozen murine colons embedded in OCT were prepared. Tissues have been fixed with 4% formaldehyde. After three washes (3 × 10 min) in PBS plus 0.5% Triton X-100 and 1% BSA, slides were incubated overnight in a humid chamber with primary antibodies in PBS-Triton X-100-BSA. After three washes in PBS-Triton X-100-BSA, slides were then incubated with appropriated secondary fluorescent-coupled antibodies for 2 h at room temperature. After wash in PBS, actin staining was performed by adding Acti-stain^TM^ 670 (Cytoskeleton, Inc.) for 30 min. Slides were finally washed three times in PBS, mounted in Prolong Gold-DAPI (Invitrogen Molecular Probes), and analyzed by confocal laser scanning using Zeiss LSM710 (Leica Microsystems, Heerbrugg, Germany).

For immunocytostaining, colonoids were seeded in eight-well Lab-Tek and fixed in 2% paraformaldehyde (20 min), washed three times in PBS (15 min), and then permeabilized in PBS with 0.5% Triton X-100 (20 min). After two washes in PBS with 100 mM glycine (20 min), blocking solution (7.7 mM NaN_3_, 1% BSA, 0.2% Triton X-100, and 0.05% Tween-20, in PBS) was added for 90 min. Primary antibody was incubated overnight at 4 °C. After three washes in blocking solution (15 min), secondary antibody was incubated for 45 min. Actin staining was performed by adding Acti-stain^TM^ 670 for 30 min followed by three washes in PBS before mounting. Then, after washes in PBS, slides were mounted in ProGold DAPI and observed by confocal laser scanning (Zeiss LSM710).

For each staining, controls were made in the same conditions with no antibody, secondary antibody only, isotype control or pre-immune serum, and staining in PAR_2_ KO tissue.

### Western blotting

Male- or female-derived six wells of colonoid culture (48-well plates) were dissociated from Matrigel by incubation with Cell Recovery Solution as described above for passage. Then, colonoids were centrifuged (43*g*, 10 min) and lysed in Laemmli sample buffer 5x. After boiling for 10 min, proteins were resolved on polyacrylamide SDS gels (SDS-PAGE) and transferred to nitrocellulose (membrane Hybond C-super, Merck Millipore). The membrane was blocked for 1 h at room temperature in Tris-buffer saline (TBS) containing 0.5% fat-free milk and 1% bovine serum albumin (BSA, Sigma). Then, membrane was probed overnight at 4 °C with the appropriate antibody in TBS-milk-BSA supplemented with 0.05% Tween. After incubation for 1 h at room temperature with secondary antibody coupled to horseradish peroxidase, detection was achieved using a chemiluminescent substrate (Amersham ECL Prime detection reagent) and visualized on ChemiDoc (Bio-Rad).

### Cell sorting

Male or female crypts were isolated by 75 min orbital shaking at room temperature of 10 washed murine colons (2/3 ends of descendant colon) in PBS with 9 mM EDTA plus 3 mM dithiothreitol and 10 μM Y-27632, followed by manual shaking for 2 min in PBS with 10 μM Y-27632. Then, the crypt suspension (around 2 × 10^5^ male crypts and 2.8 × 10^5^ female crypts) was filtered through a 100-μm cell strainer and centrifuged (40*g*, 5 min, 4 °C). Individual epithelial cells were obtained after incubation of crypts at 37 °C with dispase (60,000 units/ml, BD Biosciences) and DNase I (20,000 units/ml, Sigma) for 4 min and shaking for 30 s. The suspension of individual cells (around 1.2 × 10^6^ male cells and 0.6 × 10^6^ female cells) was filtered through a 40-μm cell strainer in 1 ml of cold FCS. After centrifugation (1000*g*, 5 min, 4 °C), cells were suspended in DMEM F12 supplemented with 100 U/ml penicillin/streptomycin, 10 mM Hepes, 2 mM Glutamax, N2 (1/100), B27 (1/50), and *N*-acetylcysteine (NAC, 1 mM, Sigma).

For cell sorting, 9 × 10^5^ male and 3 × 10^5^ female cells were labeled for 45 min at 4 °C with antibodies from BD Biosciences: CD31-FITC, CD45-FITC, CD326-APC, CD44-BV421, and CD24-PE/CF594. Controls were incubated with above antibodies minus one or viability dye (eFluor 506, Thermo Fisher Scientific). CD326, CD44, and CD24 antibodies were used to purify colonic cells into different subsets (CD326^+^ CD44^+^ CD24^high/medium/low^) using a FACS ARIA-SORP (BD Biosciences). Sorted cells were collected in cold tubes containing 25 μl of Matrigel. Around 1.2 × 10^5^ male and 2.5 × 10^5^ female CD44^+^ CD24^+^ cells were collected and cultured as colonoids (5000 cells per well, 48-well plate) as described above.

### Statistical analysis and image/cell sorting processing

For each experiment (animals, crypts, colonoids, sorted cells), male and female were processed simultaneously. Student’s *t* (two-tailed) or ANOVA tests were used for experiment analysis. *P* values or adjusted *P* values (ANOVA) < 0.05 were considered to be significant, and the correction used for multiple comparisons is indicated on the figures. Number of colonoids and gene expression were calculated from the mean of duplicate assays in each experiment.

Apotome and confocal images were imported into the Image J software for analysis. Size of around 20 colonoids was measured in each assay. Male and female colonoid size ranges were 25–80 μm and 30–120 μm, respectively. A threshold ≥ 50 μm was taken for the study of colonoid size since significant variations between sexes and between control/treatment assays were measured at this condition. Data of Ki-67 labeling in colonoids were calculated as ratio of positive Ki-67 nuclei vs total nuclei counted in the larger diameter of colonoids whose size is representative of the male and female cultures.

Data of cell sorting were analyzed with the *FlowJo* software.

## Results

### Colonoid growth is sexually dimorphic and regulated by PAR_2_

**Fig. 1:**
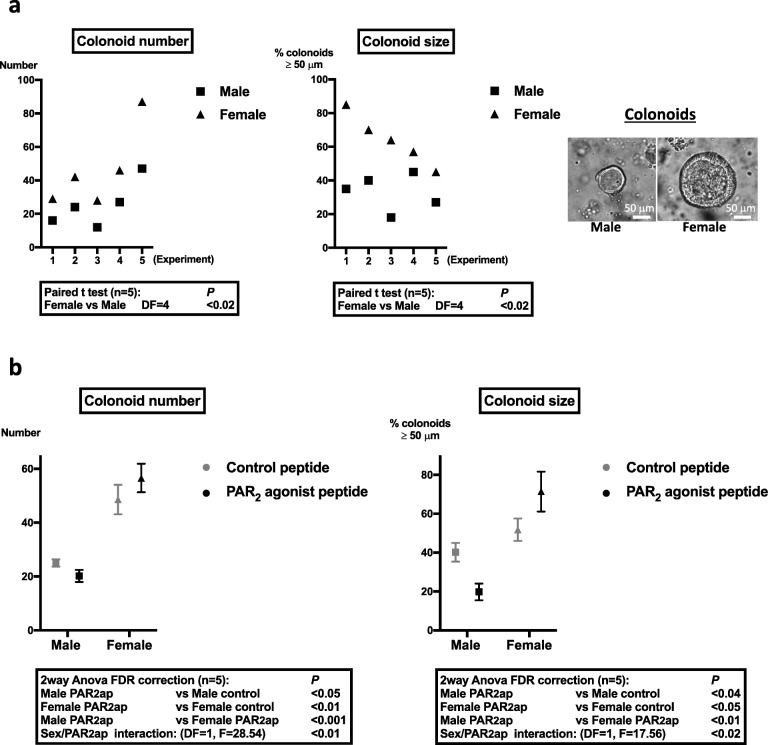
Growth characteristics of colonoids from male and female mice and impact of PAR_2_ activation. **a** Colonoids were counted and measured as described in the “Methods” section at day 6 after male and female colon crypts seeding in Matrigel. Representative colonoids are shown. **b** Colonoids from male and female mice were stimulated daily with PAR_2_ agonist peptide (SLIGRL-NH_2_, 100 μM) or control peptide (LRGILS-NH_2_, 100 μM) from day 2 to day 6 of culture. At day 6 of culture, colonoids were counted and their size measured. Results are mean ± SEM from *n* = 5 independent experiments

Colon crypts from male and female mice were embedded in Matrigel and grown as colonoids. At day 6 from initial seeding, despite identical numbers of crypts seeded, both the number and size of female mice-derived colonoids were significantly higher than those of male mice-derived colonoids (Fig. 1a). This higher size of female mice-derived colonoids was measured as soon as day 2 of culture and was maintained after re-embedding of colonoids in fresh Matrigel (Additional file 2). These data suggest that female primitive epithelial cells have higher proliferation than male.

Since we have previously shown that PAR_2_ plays a critical role in the control of ISC proliferation [7], we investigated the role of PAR_2_ in the proliferation of colon primitive cells from male and female mice. In agreement with our previous results, we measured a decrease in the number and size of colonoids from male mice treated by the PAR_2_ agonist compared to control peptide (Fig. 1b). In contrast, treatment of colonoids from female mice by the PAR_2_ agonist peptide increased their number and size compared to the control (Fig. 1b). The PAR_2_ agonist effects on colonoid growth were observed from 48 h post-treatment (Additional file 3).

Altogether, these data show that the growth of colon primitive cells is sexually dimorphic and that PAR_2_ activation further increases this difference.

### PAR_2_ controls the expression of key proliferative regulators of colon primitive cells

**Fig. 2:**
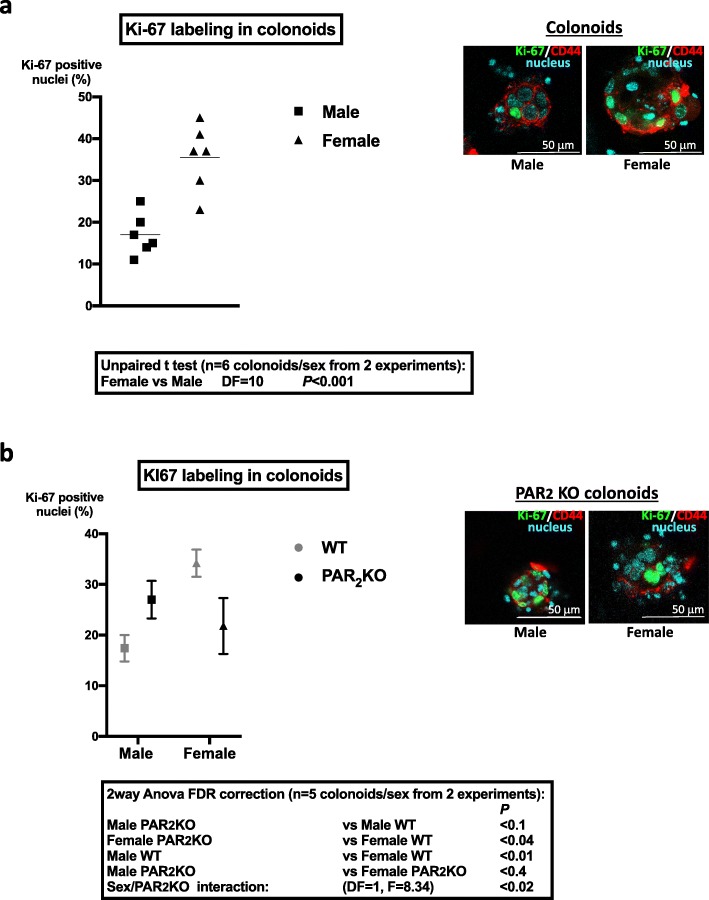
Cell proliferation in colonoids from male and female mice and impact of PAR_2_ expression. **a** Ki-67 labeling in male and female colonoids at day 6 of culture. Left panel: The percentage of Ki-67 positive nuclei was calculated as described in the “Methods” section by the ratio of positive Ki-67 nuclei vs total nuclei in the larger diameter of colonoids. Right panel: Representative colonoid labeling of Ki-67 (green), CD44 (red, immaturity marker), and nuclei by DAPI (cyan) is shown. **b** Comparative Ki-67 labeling in PAR_2_ WT and PAR_2_ KO male and female colonoids at day 6 of culture. Right panel: Representative PAR_2_ KO colonoid labeling of Ki-67 (green), CD44 (red, immaturity marker), and nuclei by DAPI (cyan) is shown. Data are mean ± SEM of 6 (**a**) or 5 (**b**) colonoids from male or female mice from *n* = 2 independent experiments

In order to evaluate the impact of PAR_2_ on colonoid growth, crypts from colons of PAR_2_ KO mice were isolated. The absence of PAR_2_ impaired colonoid culture from both male and female mice (Additional file 4) as we have previously shown that PAR_2_ is implicated in the ISC survival [7]. However, labeling of cell proliferation marker Ki-67 was performed in surviving PAR_2_-deficient colonoids and WT colonoids. Whereas Ki-67 was expressed in a higher number of cells from female WT mice compared to males (Fig. 2a), PAR_2_ KO female-derived colonoids showed decreased Ki-67 labeling compared to WT (Fig. 2b). Conversely, PAR_2_ KO male-derived colonoids showed a tendency to increase Ki-67 labeling compared to WT (Fig. 2b). As a consequence, the percentages of Ki-67 positive nuclei were not different between PAR_2_ KO colonoids from both sexes (Fig. 2b). These results show that colonoids from female WT mice contain a higher number of proliferative primitive cells (stem cells and progenitors) compared to WT males and suggest that PAR_2_ may play a critical role in that sexual dimorphism.

To investigate the role of PAR_2_ in the regulation of male and female colon primitive cell proliferation, we analyzed the gene expression of key regulators of the Wnt, Notch, and EGF proliferative pathways, as well as PAR_2_, in male and female colon crypts from WT or PAR_2_ KO mice. The gene expression of PAR_1_ and some adhesion receptors was also studied given their implication in the regulation of colon primitive cells [7].

**Fig. 3:**
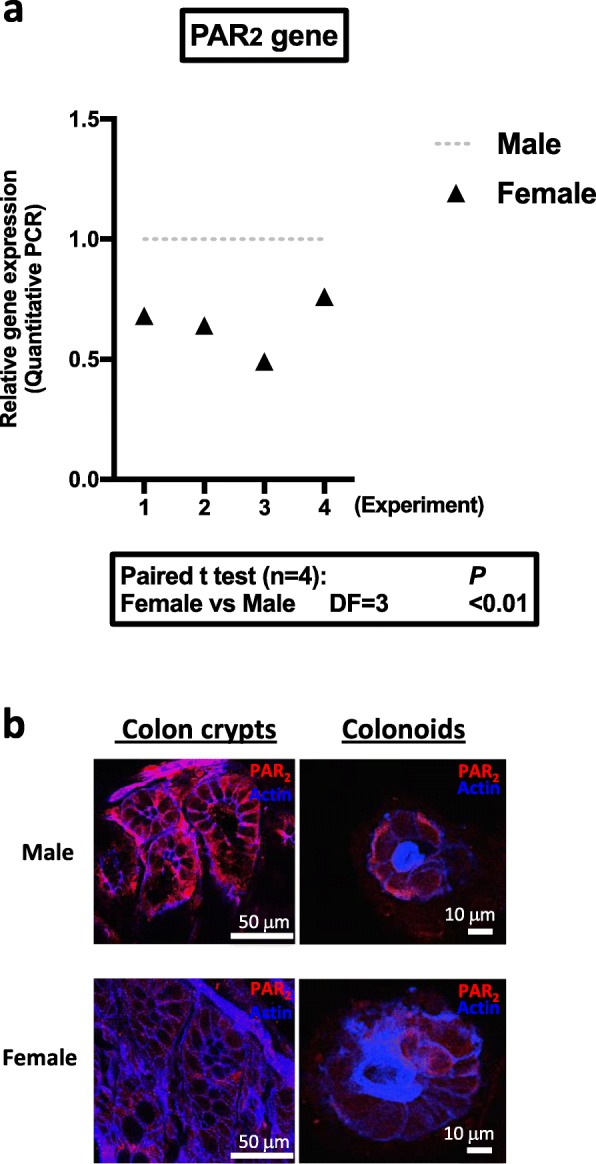
PAR expression in colon crypts and colonoids. **a** PAR_2_ mRNA expression in colon crypts from male or female mice was measured by qRT-PCR (*n* = 4 independent experiments). **b** Immunolabeling of PAR_2_ (red) in native colon crypts and cultured colonoids (day 6 of culture) from male and female mice. Actin (blue) was labeled by phalloidin. Results are representative of three independent experiments

As shown in Fig. 3a, quantitative RT-PCR detected a lower level of PAR_2_ (*F2rl1*) mRNA in colon crypts from female WT mice compared to males, whereas PAR_1_ (*F2r*) was not differentially expressed (Additional file 5, *n* = 4, DF = 3, paired *t* test *p* < 0.2). In PAR_2_ KO crypts, PAR_1_ mRNA expression did not vary significantly between males and females (Additional file 5, *n* = 4, DF = 3, paired *t* test *p* < 0.9). In the absence of suitable PAR_2_ antibody for quantification by Western blot, analysis of PAR_2_ protein expression by immunostaining in colon crypts and colonoids confirmed the lower expression in females compared to males (Fig. 3b). These data demonstrate that PAR_2_ is differently expressed in male and female colon epithelial cells.

**Fig. 4:**
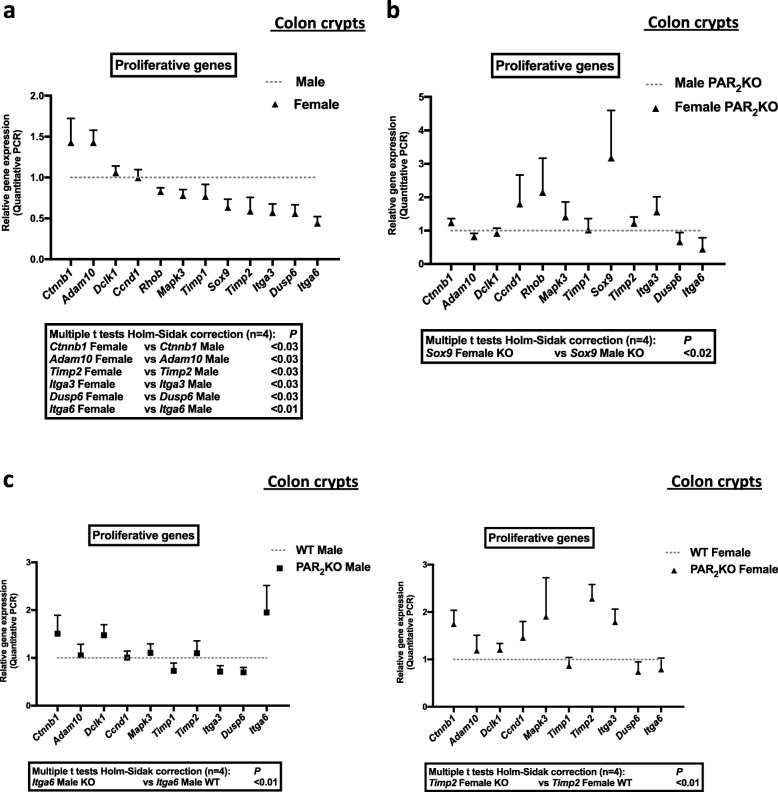
Expression of proliferative signals for colon primitive cells in PAR_2_ WT or KO male and female crypts. mRNA of male and female WT or PAR_2_ KO crypts were extracted, and the expression of key proliferative signals for colon primitive cells and their modulators was quantified by RT-PCR. **a** Comparative data from male and female PAR_2_ WT crypts. **b** Comparative data from male and female PAR_2_ KO crypts. **c** Comparative data from male PAR_2_ KO crypts vs male PAR_2_ WT crypts (left panel), and female PAR_2_ KO crypts vs female PAR_2_ WT crypts (right panel). Data are mean ± SEM from *n* = 4 independent experiments

The expression of the proliferation enhancers, *Ctnnb1* (β catenin, Wnt pathway) and *Adam10* (Disintegrin and metalloprotease 10, Notch pathway), was higher in colon crypts from female mice compared to males (Fig. 4a and Additional file 5). In contrast, the expression of the proliferation inhibitors, *Timp2* (tissue inhibitor of metalloproteinases 2, EGF pathway) and *Dusp6* (dual specificity phosphatase 6, Erk pathway), was lower in colon crypts from female mice compared to males (Fig. 4a and Additional file 5). Importantly, other modulators of colon cell proliferation, the integrins alpha 6 (*Itga6*) and alpha 3 (*Itga3*), were expressed at a higher level in colon crypts from male mice compared to females (Fig. 4a and Additional file 5). These data suggest that pathways important for ISC and progenitors proliferation (Wnt, Notch, EGF, integrins) are differently regulated in colon crypts from male and female mice.

In PAR_2_ KO crypts, the sexual dimorphism in the expression of *Ctnnb1* (β catenin), *Adam10*, *Timp2*, *Itga3*, *Dusp6*, and *Itga6* was abolished (Fig. 4b and Additional file 5). Interestingly, the expression of *Sox9*, a transcriptional factor playing a key role in male sex determination and stem cell proliferation, was reversed compared to WT since it was higher in PAR_2_ KO female-derived crypts compared to PAR_2_ KO males (Fig. 4b and Additional file 5). *Sox9* varied in female PAR_2_ KO (6.60 ± 5.60 mean ± SD, fold increase vs WT) but not in male PAR_2_ KO (0.85 ± 0.14 mean ± SD, fold increase vs WT). Analysis of significant variations of genes revealed that *Itga6* in males and *Timp2* in females were under the control of PAR_2_ (Fig. 4c and Additional file 5). Thus, in the absence of PAR_2_, the basal sexual dimorphism in *Itga6* expression was reinforced, whereas *Timp2* was specifically upregulated in females. These data show that PAR_2_ controls gene expression of important regulators of the ISC and progenitor proliferation.

Altogether, our data suggest that PAR_2_ may play a specific and critical role in the control of proliferation in colon crypts from male and female mice.

### The sexual dimorphism in colonoid growth is linked to metabolic and resistant phenotypes

We previously demonstrated the PAR_2_-dependent regulation of the glycogen synthase kinase 3 (GSK3) in ISC [7]. Given the critical role of GSK3 to promote quiescence and survival of primitive cells [4, 7], we investigated its expression and activation in colons from male and female mice.

**Fig. 5:**
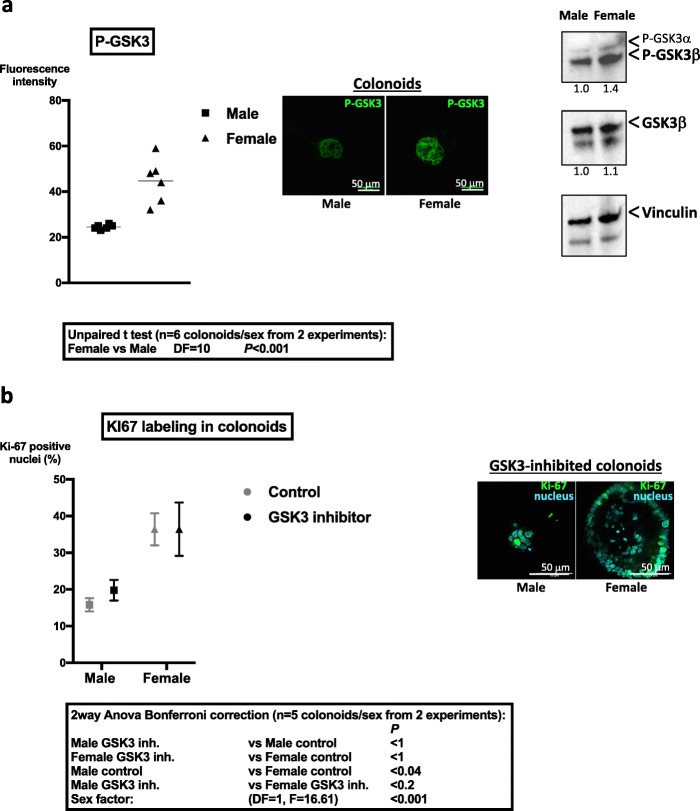
Differential regulation of GSK3 in colon primitive cells from male and female mice. **a** Immunolabeling of Pser21/9 GSK3 in colonoids from male and female mice was performed at day 6 of culture. Fluorescence intensity of the brightest cross section of colonoids was quantified by Image J and is represented (*n* = 2 independent experiments). Representative images of immunolabeling of PserGSK3 are shown. Right panel: Western blot of Pser21/9 GSK3 and total GSK3β in colonoids is shown. Vinculin was used as loading control. Results are representative of two independent experiments. **b** Colonoids from male and female mice were incubated daily with GSK3 inhibitor (SB216763, 12.5 μM) from day 2 to day 6 of culture. Ki-67 labeling in male and female colonoids treated by GSK3 inhibitor was performed at day 6 of culture. The percentage of Ki-67 positive nuclei was calculated as described in the “Methods” section by the ratio of positive Ki-67 nuclei vs total nuclei in the larger diameter of colonoids. Representative colonoid labeling of Ki-67 (green) and nuclei by DAPI (cyan) is shown. Data are from two independent experiments

No significant difference in the mRNA expression of GSK3 (β isoform) between sexes was measured in colon crypts (relative expression female vs male 1.16 ± 0.19, mean ± SD, *n* = 3 independent experiments). However, the inhibited form of GSK3 (Pser21/9 GSK3) was higher expressed in colonoids from female mice showing that GSK3 is more active in colonoids from male mice compared to females (Fig. 5a). Accordingly, Western blot analysis showed increased serine 9 phosphorylated-GSK3β in female-derived colonoids compared to males (Fig. 5a).

Incubation with the GSK3 inhibitor SB216763 induced a specific decrease of the number of colonoids from male mice compared to control (*n* = 4; male − 47% ± 11, mean ± SD, two-way ANOVA *p* < 0.001; female + 13% ± 26%, mean ± SD, two-way ANOVA ns). Moreover, this treatment abolished the difference in Ki-67 labeling between colonoids from both sexes (Fig. 5b). Thus, the kinase GSK3 may be implicated in the sexual dimorphism of colonoid growth.

**Fig. 6:**
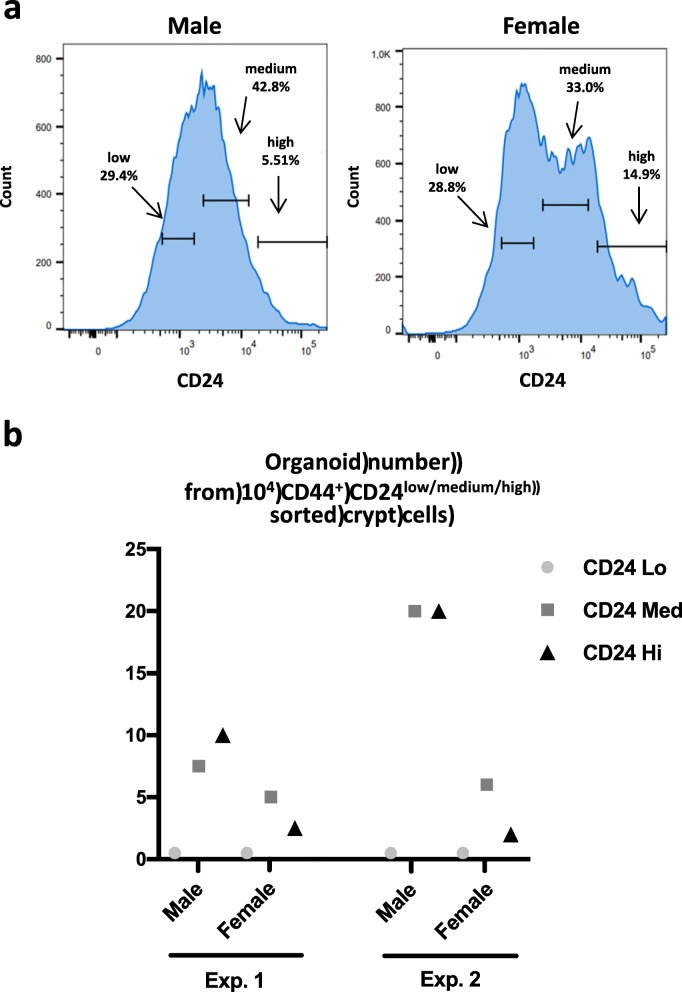
**a** A cell sorting of CD44^+^ CD24^+^ primitive cells was performed from male and female colon crypts. Three CD24 subpopulations were isolated as defined on the graphs (representative of three experiments). The percentage of each CD24 subpopulation is shown. **b** Sorted CD44^+^ CD24^high/medium/low^ were embedded in Matrigel for colonoid culture. At day 10, colonoids were counted. Data are from *n* = 2 independent experiments in duplicate

The above data suggest that proliferation and survival of colon primitive cells from male and female mice might be under the control of different metabolic pathways. Thus, we evaluated their growth ability under high stress conditions such as cell sorting. The adhesion molecules CD44 and CD24 are markers of the colon primitive cells and can be used for cell sorting protocols presenting the advantages to collect both slowly proliferative/strongly resistant ISC and highly proliferative/poorly resistant ISC [23, 24]. Note that *Cd44* and *Cd24* gene expressions were not significantly different between male and female colon crypts (male vs female *n* = 4, two-way ANOVA CD24 *p* < 0.3 and CD44 *p* < 0.9; Additional file 5). The cell sorting of colon primitive cells based on CD44 and CD24 allowed the collection of three cell populations CD44^+^CD24^low^, CD44^+^CD24^medium^, and CD44^+^CD24^high^ (Fig. 6a). Note that the fraction of CD44^+^CD24^high^ cells was constantly higher in females (percentage of CD44^+^CD24^high^ cells vs entire CD44^+^CD24^+^ population: male 7.1 ± 2.3%, female 13 ± 3.2%, mean ± SD, *n* = 3, paired *t* test *p* = 0.05). After embedding in Matrigel, only the CD44^+^CD24^medium^ and CD44^+^CD24^high^ cells developed as colonoids, cells from male mice showing greater efficacy (Fig. 6b). This suggests male primitive cells may be more resistant to the cell sorting process.

Altogether, these data show that male and female colon primitive cells display a sexual dimorphism in their proliferation, metabolism, and adaptation to stress, which could be critically influenced by PAR_2_.

## Discussion

This work shows that male and female colon primitive cells have different proliferation capacities and that this is controlled by the protease-activated receptor PAR_2_. As colonoids, primitive cells from female mice displayed higher proliferation compared to males. Conversely, after complete epithelial cell dissociation, primitive cells from male mice produced more colonoids than females. Furthermore, PAR_2_ was shown to control key proliferative pathways for colon primitive cells in both males and females, although in different ways.

The strong proliferative capacities of ISC and progenitors from female mice allowed the development of a higher number and size of colonoids from bottom crypts, as compared to males. This observation is in accordance with the recent data of Zhou and collaborators [25]. Moreover, this sexual dimorphism in ISC and progenitor proliferation depends on intrinsic mechanisms since the culture of epithelial cells as colonoids was exempt from stroma. Zhou and collaborators [25] also found that proliferation of intestinal organoids was not influenced by estrogens. In drosophila, Hudry and collaborators [5] demonstrated that cell-intrinsic mechanisms linked to sex determination genes control cell cycle duration in female ISC. We found that important regulators of the ISC proliferation (β catenin/Wnt pathway, ADAM10/Notch pathway) displayed gene overexpression in female-derived crypts compared to males. However, female- and male-derived crypts did not show significant differences in gene expression of immature markers CD44 and CD24 (our data) and in their size in vivo [25], suggesting sex-specific regulation of proliferative and differentiation pathways at the progenitor level.

The crypt microenvironment is shaped by proteases through matrix proteolysis, release of growth factors, and receptor activation. The Wnt and Notch pathways are tightly controlled by this microenvironment. It is also true for the EGF pathway, a key regulator of the progenitor proliferation and differentiation [26], and our data show gene overexpression of inhibitors of this pathway (*Dusp6*, *Timp2*) in crypts from male mice compared to females. We have shown that the protease-activated receptor PAR_2_ was expressed in epithelial cells along the male and female crypts, but at a higher level in males. PAR_2_ was found required for survival of colonoids from both male and female mice and supports sexual dimorphism in the expression of proliferative genes in the crypt. Thus, depending on its level of expression in the crypt, PAR_2_ could promote the sexual dimorphism in the proliferation of colon primitive cells.

However, PAR_2_ activation had an opposite impact on growth of colonoids from male and female mice. PAR_2_ slows down the growth of colonoids from male mice but increases it from females. The growth of male-derived colonoids is associated with an active status of the kinase GSK3, a key modulator of cell metabolism and proliferation [27]. We have previously shown that the activation of GSK3 was under the control of PAR_2_ in colonoids from male mice [7]. It is therefore likely that the regulation of GSK3 represents a critical point in the sexual dimorphism of ISC function, as we previously demonstrated for leukemic stem cells [4]. Finally, a different subcellular localization of PAR_2_ in intestinal epithelial cells between male and female may also reflect differential presence of PAR_2_-activating proteases in the epithelial microenvironment [28]. Further studies would be necessary to investigate potential differential expression and activity of intestinal proteases in males and females.

The knockout of PAR_2_ abolished the sexual dimorphism in ISC proliferation and in gene expression of crypts described above. The protease-activated receptor PAR_1_ is co-expressed with PAR_2_ in epithelial cells, and we demonstrated that it oppositely regulates colonoid growth and GSK3 compared to PAR_2_ [7]. PAR_1_ was not differently expressed at gene level in male and female crypts, WT or PAR_2_ KO. This demonstrates that PAR_2_, but not PAR_1_, is critical for the sexual dimorphism in ISC function. Compared to WT, the gene expression of α_6_ integrin increased strongly in crypts from PAR_2_ KO male mice. Since the cellular balance between α_6_A and α_6_B variants influence cell proliferation in the crypt [29], it is possible that the proliferative isoform A of α_6_ integrin was differentially expressed in PAR_2_ KO crypts. Moreover, we measured an increase of gene expression of TIMP2 in crypts from PAR_2_ KO female mice compared to WT. Interestingly, TIMP2 has been described to directly bind to α_3_ integrin (*Itga3* also increased in PAR_2_ KO female-derived crypts) in a context of growth arrest [30]. Furthermore, *Timp2*, *Itga3*, and *Sox9* which increased in PAR_2_ KO females are located on the same chromosome (17 human, 11 murine) where there is controlled sex reversal [31]. As demonstrated by Hudry and coll. in drosophila [5], crypt stem cells have to undergo sex reversal-related plasticity in physiological or pathological situations. A potential role of PAR_2_ in that process should be investigated. Altogether, our results demonstrate that PAR_2_ controls the proliferation of both male and female colon primitive cells, in opposite ways, and possibly their plasticity.

An interesting observation was that both PAR_2_-deficient male and female colon primitive cells become vulnerable in stressed conditions such as in vitro culture. We have shown previously that the PAR_2_/GSK3 pathway is critical in the control of male colon primitive cell survival [7]. Moreover, integrins are key partners for PAR_2_ and GSK3 to control cell survival [4, 32], and we previously demonstrated that cell adhesion mechanisms related to the Rho kinase activation influenced the PAR_2_/GSK3 pathway in colon primitive cells [7]. Thus, it is possible that primitive cells in the male colon crypt have better adhesion capacities and resistance to stress leading to increased cell survival compared to females. In favor of this hypothesis are the lesser efficacy of the male crypt extraction and the better growth of sorted male CD24^high/med^ epithelial cells, compared to females. However, we measured great variations in the efficiency of male colonoid growth from sorted cells between the two experiments shown. Following a sorting protocol closed to ours, Yip and collaborators [24] have also obtained important variations in organoid growth from male mice despite the presence of myofibroblastic growth factors in the medium culture that enhance survival and proliferation of primitive cells. This suggests that during the cell sorting process, important mechanisms aiming cells to cope with de-adhesion stress are set up. However, this setting-up, involving probably PAR_2_ engagement which protects male colon epithelial cells from anoïkis [7], might display variable efficacy depending on dispase incubation for the isolation of individual cells. Interestingly, dispase has been shown to induce a specific internalization of the alpha 6 integrin [33], which is overexpressed in male colon epithelial cells compared to females and whose expression is under the control of PAR_2_. Knowing that resistant primitive cells in the crypt are in the vicinity of progenitors, specific sex-dependent mechanisms could be critical in that zone. The lower proliferation rate of male progenitors associated with their specific metabolism may provide an advantage for survival to stress compared to females. This observation may have important pathophysiological implications in intestinal inflammation and cancer displaying a sexual dimorphism in incidence and location [10, 11].

## Perspectives and significance

**Fig. 7:**
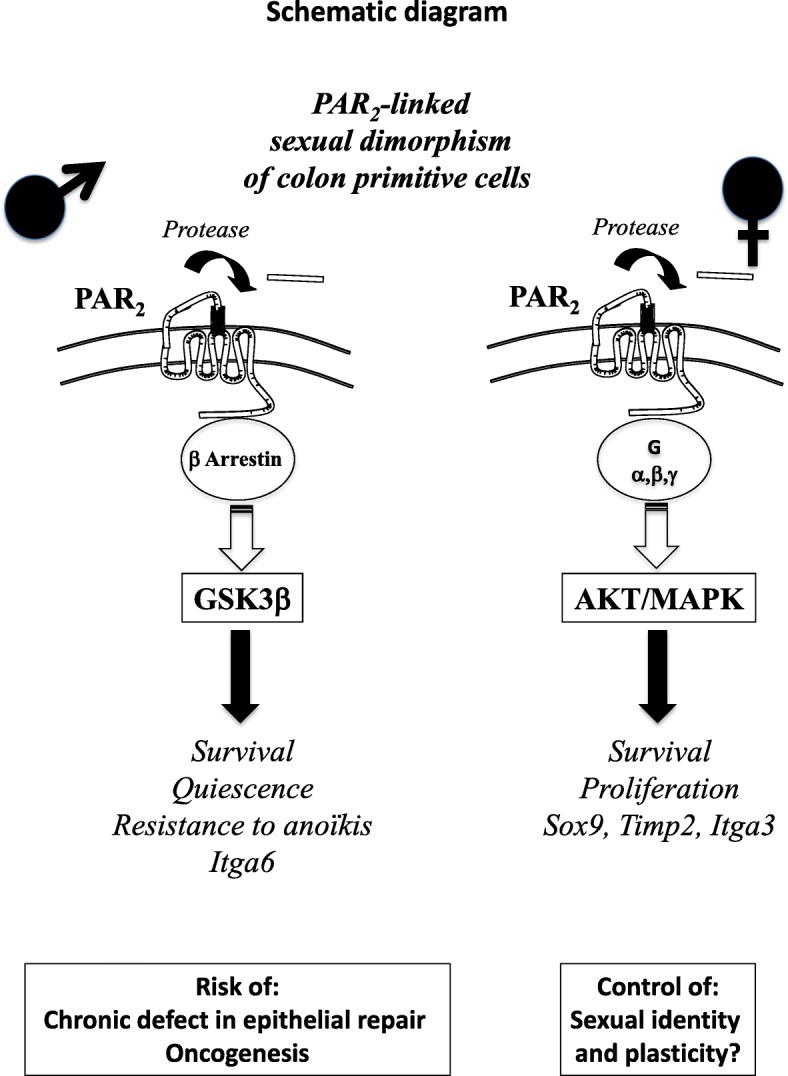
Sexual dimorphism occurs in the PAR2-dependent regulation of colon primitive cells, which could have important implications in pathophysiology and therapy

A sexual dimorphism has already been shown in PAR_2_ functions with increased PAR_2_-mediated vasodilatation and pruritus associated with female sex. Our data show now that a sexual dimorphism occurs in the PAR_2_-dependent regulation of colon primitive cells, which could have important implications in pathophysiology and therapy (Fig. 7). Indeed, compared to females, the higher capacity of male-derived progenitor/stem cell coping with stress to develop PAR_2_-dependent survival coupled with quiescence may be first advantageous but can become deleterious during repeated aggressions such as chronic inflammation. As a consequence, there is a risk of chronic defect in epithelial repair and accumulation of mutations paving the way for oncogenesis. A crosstalk between integrins such as integrin α6 and PAR_2_ may play a critical role in that context. On the other hand, we show that PAR_2_ controls the expression of genes implicated in sexual dimorphism such as *Sox9* and *Timp2* suggesting that PAR_2_ may be an important regulator of stem cell sexual identity and plasticity.

## Additional files

Additional file 1: Colonoid culture with or without phenol red. (DOCX 52 kb)

Additional file 2: Time course of colonoid culture and passage. (DOCX 54 kb)

Additional file 3: Time course of PAR_2_-stimulated colonoid culture. (PPTX 210 kb)

Additional file 4: Time course of PAR_2_ KO colonoid culture. (DOCX 57 kb)

Additional file 5: DeltaCt of proliferative genes. (DOCX 72 kb)

## Data Availability

The datasets generated and analyzed during the current study are available from the corresponding author on reasonable request.

## Abbreviations

ADAM10: Disintegrin and metalloprotease 10
DCLK1: Doublecortin-like kinase 1
DUSP6: Dual specificity phosphatase 6
EGF: Epidermal growth factor
GSK3: Glycogen synthase kinase 3
ISC: Intestinal stem cell
NAC: *N*-Acetylcysteine
PAR: Protease-activated receptor
TIMP: Tissue inhibitor of metalloproteinases
