# Supplementary figures and images for: Sexual dimorphism in PAR2-dependent regulation of primitive colonic cells
Source: Biol Sex Differ. 2019 Sep 6;10:47. doi: 10.1186/s13293-019-0262-6 (PMC6731565; doi:10.1186/s13293-019-0262-6)

## Slide 1
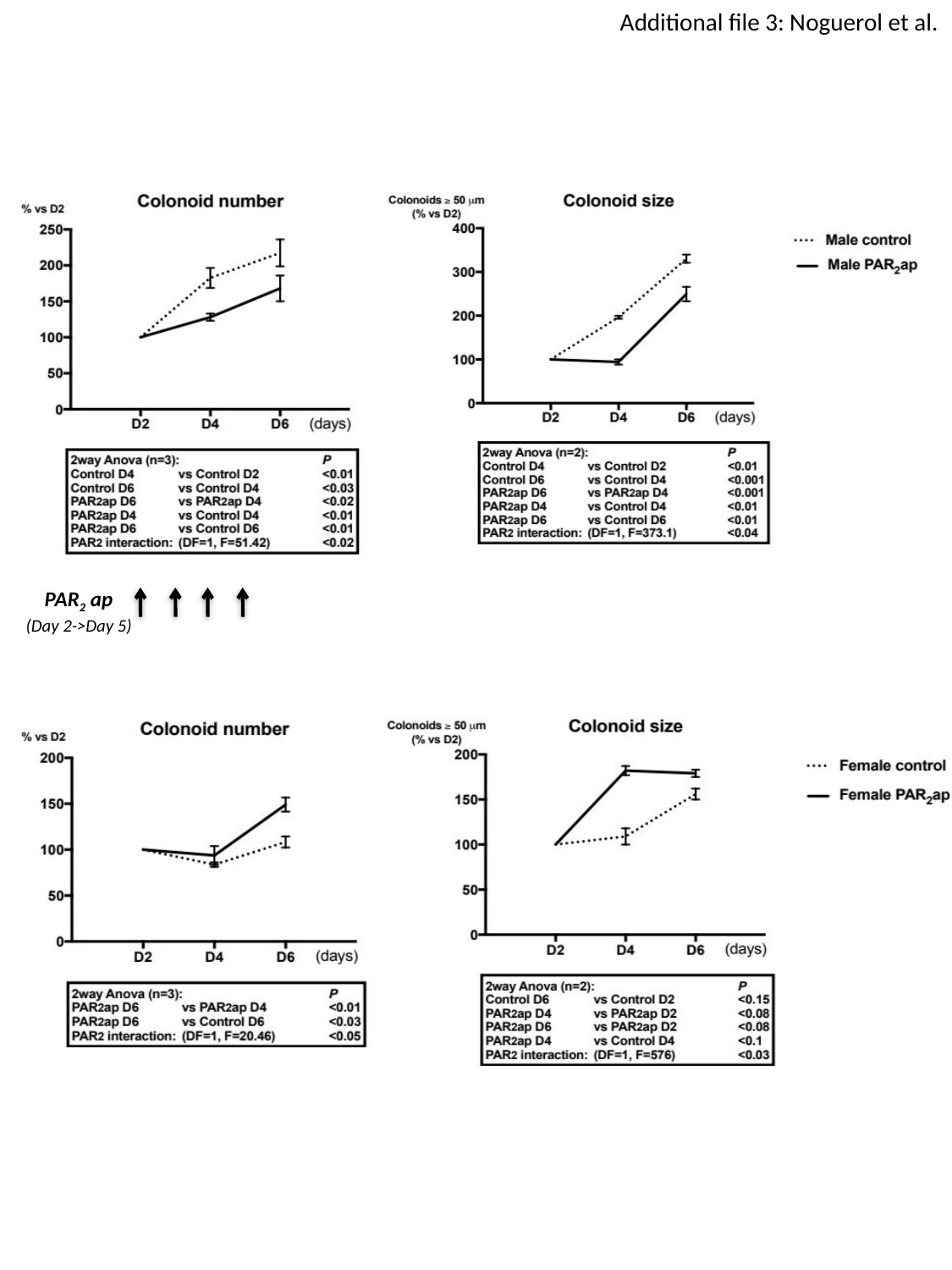

Additional file 3: Noguerol et al.
PAR2 ap
(Day 2->Day 5)

Supplement: Supplementary file 3 — Time course of PAR2-stimulated colonoid culture. (PPTX 210 kb) [file 13293_2019_262_MOESM3_ESM.pptx]
