# Supplementary material for: Sexual dimorphism in PAR2-dependent regulation of primitive colonic cells
Source: Biol Sex Differ. 2019 Sep 6;10:47. doi: 10.1186/s13293-019-0262-6 (PMC6731565; doi:10.1186/s13293-019-0262-6)
Supplement: Supplementary file 2 [file 13293_2019_262_MOESM1_ESM.docx]

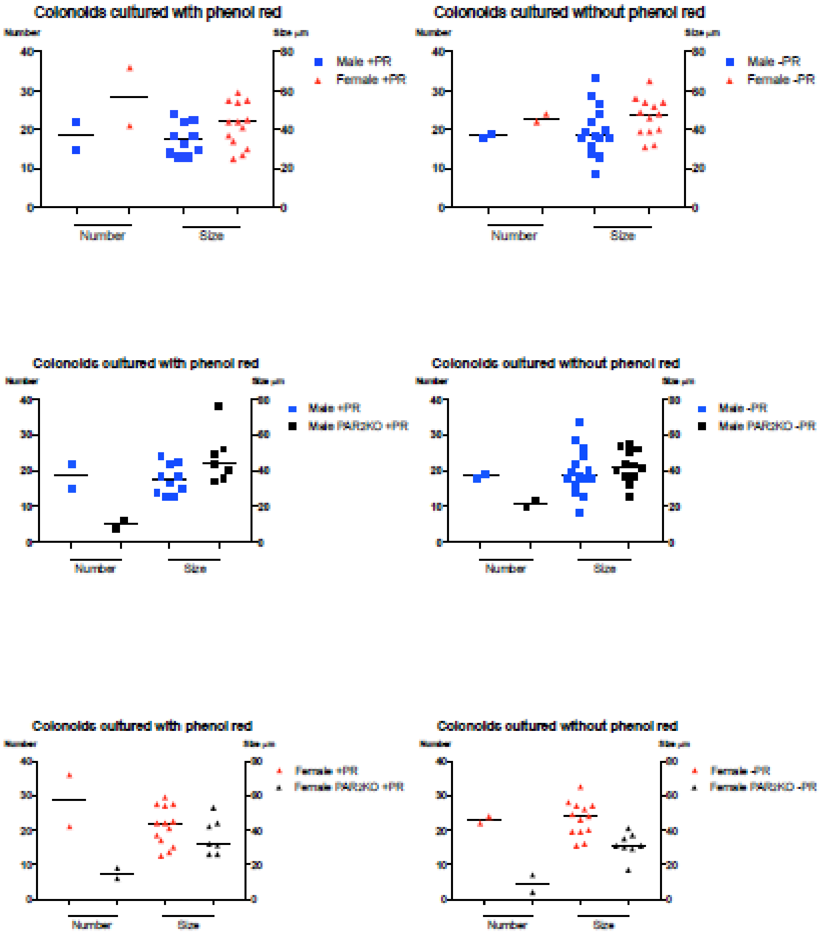
Colonoids cultured with phenol red
Number
Size µm
Male +PR
Female +PR
Number
Size
Colonoids cultured without phenol red
Male -PR
Female -PR
Colonoids cultured with phenol red
Male +PR
Male PAR2KO +PR
Colonoids cultured without phenol red
Male -PR
Male PAR2KO -PR
Colonoids cultured with phenol red
Female +PR
Female PAR2KO +PR
Colonoids cultured without phenol red
Female -PR
Female PAR2KO -PR
