# Supplementary material for: Sexual dimorphism in PAR2-dependent regulation of primitive colonic cells
Source: Biol Sex Differ. 2019 Sep 6;10:47. doi: 10.1186/s13293-019-0262-6 (PMC6731565; doi:10.1186/s13293-019-0262-6)
Supplement: Supplementary file 4 [file 13293_2019_262_MOESM4_ESM.docx]

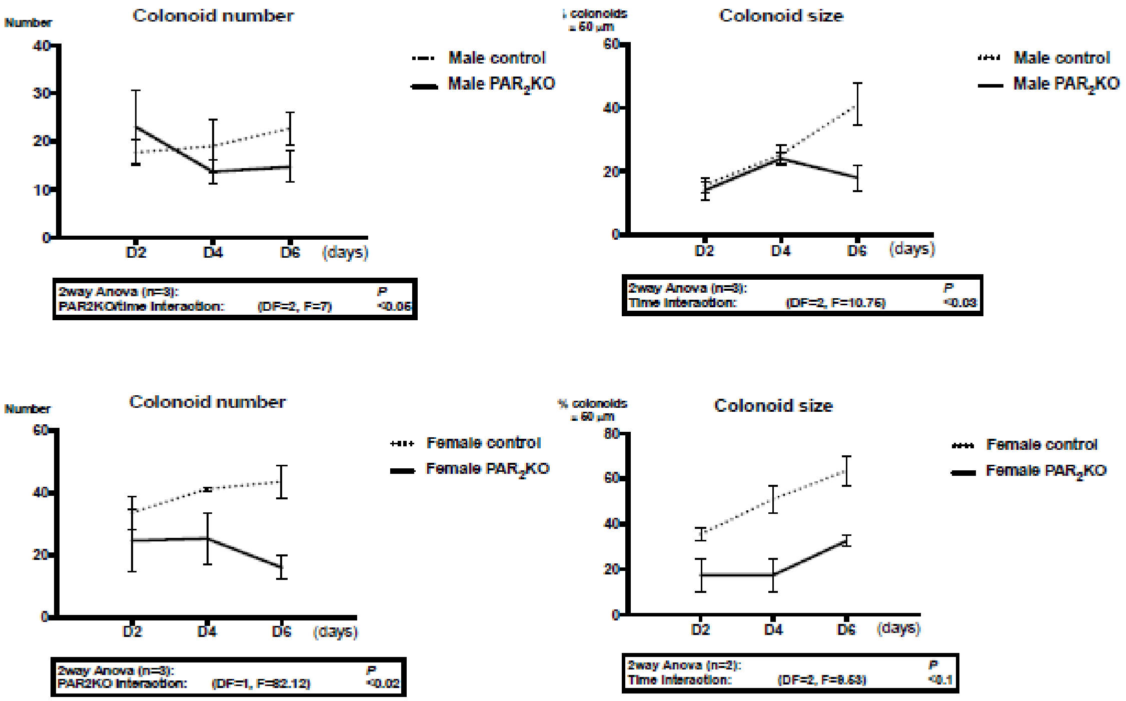
Colonoid number
Number
40
30
20
10
0
D2
D4
D6
(days)
Male control
Male PAR_2KO
2way Anova (n=3): P
PAR2KO/time interaction: (DF=2, F=7) <0.05
Colonoid size
% colonoids ≥ 60 µm
60
40
20
0
D2
D4
D6
(days)
Male control
Male PAR_2KO
2way Anova (n=3): P
Time interaction: (DF=2, F=10.75) <0.03
Colonoid number
Number
60
40
20
0
D2
D4
D6
(days)
Female control
Female PAR_2KO
2way Anova (n=3): P
PAR2KO interaction: (DF=1, F=82.12) <0.02
Colonoid size
% colonoids ≥ 60 µm
80
60
40
20
0
D2
D4
D6
(days)
Female control
Female PAR_2KO
2way Anova (n=2): P
Time interaction: (DF=2, F=8.53) <0.1
