# Supplementary material for: Sexual dimorphism in PAR2-dependent regulation of primitive colonic cells
Source: Biol Sex Differ. 2019 Sep 6;10:47. doi: 10.1186/s13293-019-0262-6 (PMC6731565; doi:10.1186/s13293-019-0262-6)

Additional file 5: Noguerol et al.

Murine colon crypts n=4

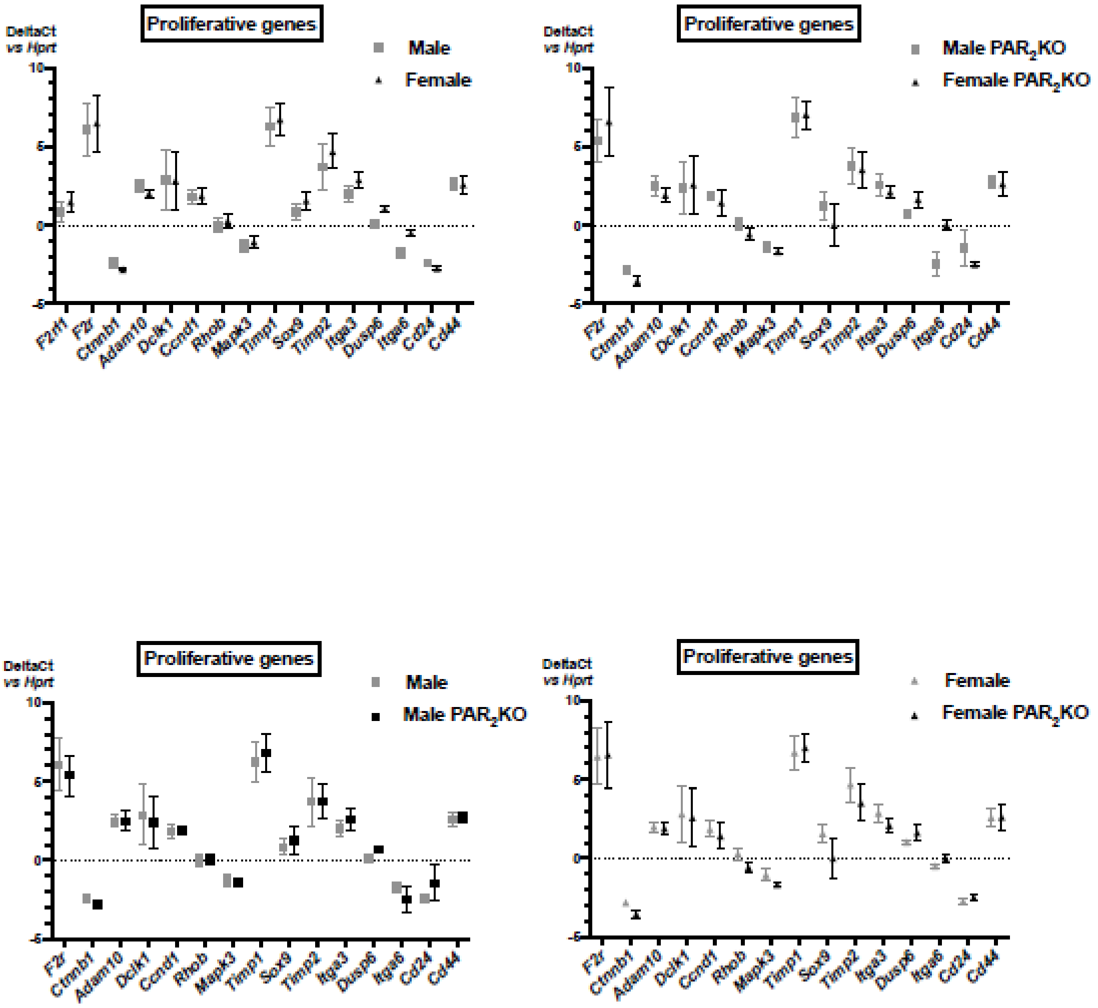

Supplement: Supplementary file 5 [file 13293_2019_262_MOESM5_ESM.docx]
